# Inhibition of PTEN activity aggravates cisplatin-induced acute kidney injury

**DOI:** 10.18632/oncotarget.20790

**Published:** 2017-09-08

**Authors:** Jun Zhou, Youling Fan, Simin Tang, Huiping Wu, Jiying Zhong, Zhengxing Huang, Chengxiang Yang, Hongtao Chen

**Affiliations:** ^1^ Department of Anesthesiology, The First People’s Hospital of Foshan, Foshan, Guangdong Province, 528000, China; ^2^ Department of Anesthesiology, Panyu Central Hospital, Guangzhou, Guangdong Province, 511400, China; ^3^ Department of Anesthesiology, Eighth People’s Hospital of Guangzhou, Guangzhou, Guangdong Province, 510060, China

**Keywords:** acute kidney injury, PTEN, inflammatory cytokines, apoptosis, p53

## Abstract

Cisplatin (cis-Diamminedichloroplatinum II) has been widely and effectively used in chemotherapy against tumors. Nephrotoxicity due to cisplatin is one of the most common clinical causes of acute kidney injury (AKI), which has a poor prognosis and high mortality. The signaling mechanisms underlying cisplatin-induced AKI are not completely understood. Phosphatase and tensin homolog deleted on chromosome 10 (PTEN) is a tumor suppressor that negatively regulates the cell-survival pathway and is considered a double-edged sword in organ damage. In this study, we examined the effect that inhibiting PTEN activity in experimental models of cisplatin-induced AKI had on the degrees of AKI. Compared with vehicle mice, mice treated with bpV(pic) (specific inhibitor of PTEN) had exacerbated renal damage due to cisplatin-induced AKI. Furthermore, inhibition of PTEN activity increased cell apoptosis in the kidneys of mice induced by cisplatin. More inflammatory cytokines were activated after cisplatin treatment in mice of the bpV(pic)-treated group compared with vehicle mice, and these inflammatory cytokines may be partially derived from bone marrow cells. In addition, inhibiting PTEN activity decreased the phosphorylation of p53 in the pathogenesis of cisplatin-induced AKI. In summary, our study has demonstrated that inhibiting PTEN activity aggravates cisplatin-induced AKI via apoptosis, inflammatory reaction, and p53 signaling pathway. These results indicated that PTEN may serve as a novel therapeutic target for cisplatin-induced AKI.

## INTRODUCTION

Acute kidney injury (AKI) is a primary cause of renal failure and a serious global health concern [1]. New strategies for the prevention and treatment of AKI are needed to reduce its morbidity and mortality. Cisplatin (cis-Diamminedichloroplatinum II) can lead to AKI because of its kidney toxicity [2]. Despite improvements in the understanding of the diverse aspects related to cisplatin-induced AKI, the cellular and molecular mechanisms are not fully understood. A better understanding of the pathogenesis of cisplatin-induced AKI is essential for developing novel therapeutic strategies to prevent its progression.

Phosphatase and tensin homolog deleted on chromosome 10 (PTEN) is not only a protein but also a lipid phosphatase that can negatively regulate the serine/threonine kinase Akt [3]. PTEN is highly expressed in multiple organ tissues, and it has been reported that PTEN inhibition protects neurons, intestines, and cardiomyocytes [4, 5, 6]. In contrast, the inhibition of PTEN has been shown to promote cyclosporine-induced nephrotoxicity [7]. Further studies have indicated that suppressing PI3K/Akt activation attenuates AKI that was induced by cisplatin [8]. However, the exact role of PTEN inhibitors in cisplatin-induced AKI is not clear.

Traditionally, PTEN is considered a negative regulator of the oncogenic PI3K/Akt signaling pathway and participates in regulating the growth of tumor-solid cancers [9]. Recent evidence indicated that the PTEN regulating PI3K/Akt signaling pathway was involved in the acute injury pathogenesis of multiple organs such as the liver and spinal cord [10, 11]. Wang YD showed that the PTEN signaling pathway participated in AKI via regulation of apoptosis [12], and Schaalan MF demonstrated that the activation of inflammatory reactions via the PTEN signaling pathway plays a crucial role in the development of AKI [13]. In addition, p53 is found to be associated with PTEN in immune regulation [14]. Thus, we hypothesized that the PTEN signaling pathway mediates cell apoptosis, inflammatory reactions and the p53 signaling pathway in cisplatin-induced AKI. Here, we investigated the role of a PTEN-specific inhibitor in murine models of cisplatin-induced AKI. Our results demonstrate that inhibiting PTEN activity via the p53 signaling pathway and aggravating cisplatin-induced AKI is involved in apoptosis and inflammatory reaction.

## RESULTS

### Effect on PTEN activity with bpV(pic)

**Figure 1:**
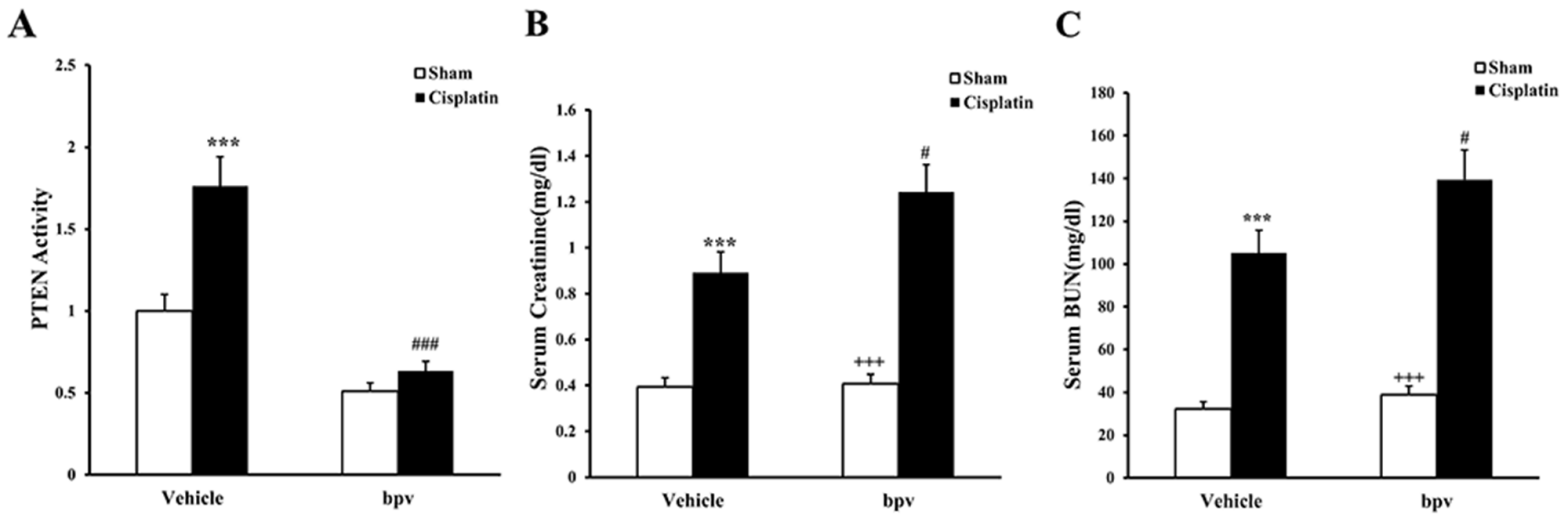
PTEN activity in renal tissue and kidney function in mice treated with cisplatin **(A)** PTEN activity in renal tissue of vehicle and bpV(pic) administrated mice after sham or cisplatin treated 72h with ELISA. ***p<0.001 vs. Vehicle Sham; ^###^p<0.001 vs. Vehicle cisplatin, n=6 each. **(B)** Effect on serum creatinine in vehicle and bpV(pic) mice at 72h after cisplatin or sham treatment. ***p<0.001 vs. Vehicle Sham; ^+++^p<0.001 vs. bpV(pic) cisplatin; ^##^p<0.01 vs. Vehicle cisplatin, n=6 each. **(C)** Effects on serum urea nitrogen in vehicle and bpV(pic) mice at 72h after cisplatin or sham treatment. ***p<0.001 vs. Vehicle Sham; ^+++^p<0.001 vs. bpV(pic) cisplatin; ^##^p<0.01 vs. Vehicle cisplatin, n=6 each.

We first determined if PTEN activity was induced in a mouse model’s kidney with cisplatin-induced AKI. Meanwhile, BALB/c mice were treated i.p. with sham or bpV(pic) at 200μg/kg once every 4h, then we detected activity of PTEN in the kidneys of mice. Using enzyme-linked immuno sorbent assay (ELISA), we found that the PTEN activity was significantly up-regulated in the kidneys of cisplatin-treated mice compared with sham controls but was significantly inhibited in the kidneys of bpV(pic)-treated mice compared with vehicle controls. (Figure 1A).

### Inhibition of PTEN activity aggravates cisplatin-induced AKI

**Figure 2:**
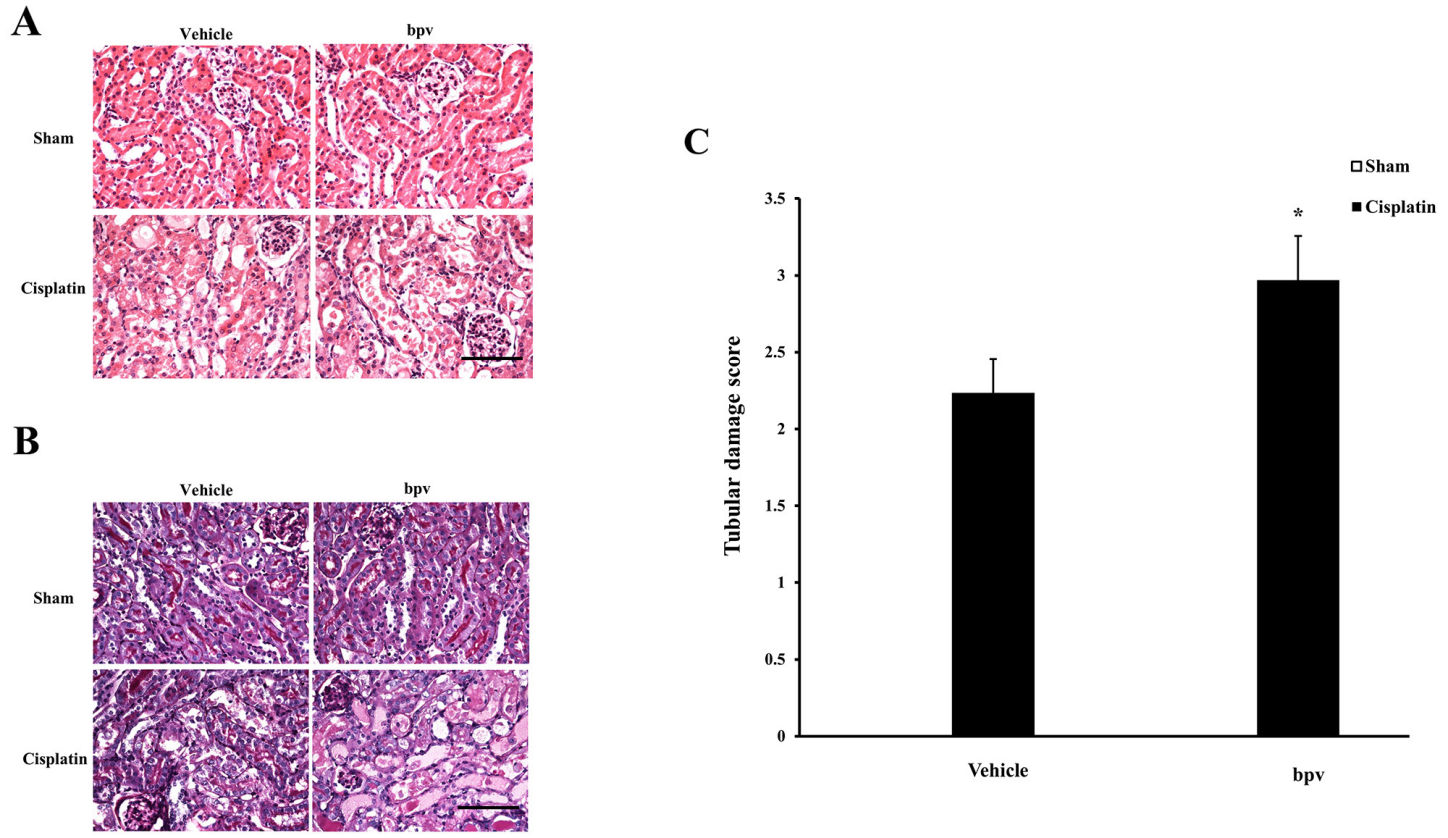
Inhibition of PTEN activity exacerbated cisplatin-induced AKI **(A)** Representative photomicrographs of HE staining for renal sections of vehicle and bpV(pic) administrated mice at 72h after cisplatin or sham treatment. (Original magnification: ×400). **(B)** Representative photomicrographs of PAS staining for renal sections of vehicle and bpV(pic) administrated mice at 72h after cisplatin or sham treatment. (Original magnification: ×400). **(C)** Quantitative assessment of tubular damage in vehicle and bpV(pic) administrated mice at 72h after cisplatin treatment according to HE staining. **P < 0.01 vs. Vehicle cisplatin. n = 6 each.

We next investigated the effect of PTEN inhibitor on the pathogenesis of cisplatin-induced AKI, BALB/c mice were treated i.p. with sham or cisplatin at 20 mg/kg. Mice developed renal dysfunction, as reflected by a marked elevation of serum creatinine and urea nitrogen 72 h after cisplatin treatment. Renal function was relatively aggravated in bpV(pic)-treated mice, with the serum creatinine and urea nitrogen levels markedly higher than those in vehicle mice (Figure 1B, 1C). Consistent with the deterioration of kidney function in bpV(pic)-treated mice following cisplatin treatment, there was a substantial augmentation in the histological injury of the kidneys, as demonstrated by more tubular epithelial cell injury, tubular dilation, and intra-tubular cast formation in bpV(pic)-treated mice (Figure 2A, 2B, and 2C).

### Inhibition of PTEN activity increases apoptotic cell death

**Figure 3:**
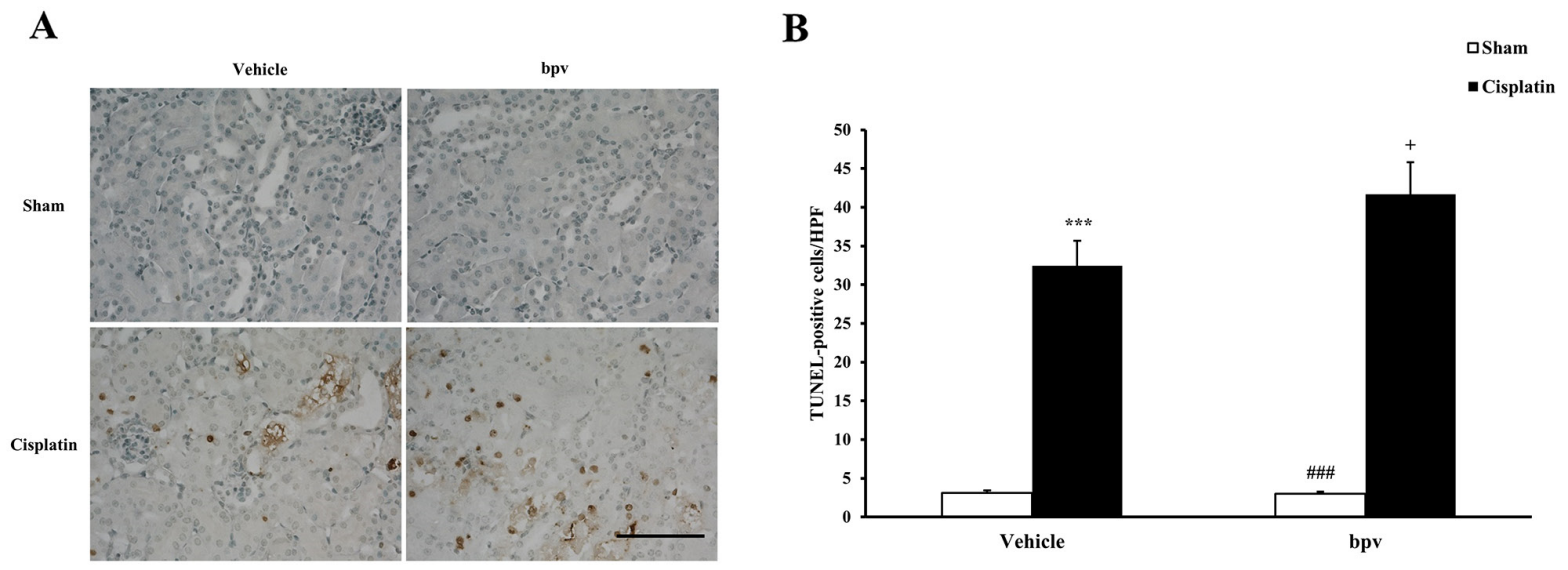
Inhibition of PTEN activity increased tubular epithelial cells from apoptosis in AKI **(A)** Representative photomicrographs of renal sections stained for apoptotic cells (brown) and counterstained with methylgreen (green) in vehicle and bpV(pic) administrated mice at 72h after cisplatin or sham treatment. (Original magnification: ×400). **(B)** Quantitative analysis of apoptotic cells in the kidneys from vehicle and bpV(pic) administrated mice after cisplatin or sham treatment. ***P < 0.001 vs. Vehicle Sham; ^+^P < 0.05 vs. Vehicle Cisplatin; ^###^P < 0.001 vs. bpV(pic) Cisplatin. n = 6 each. HPF, high power field; TUNEL, terminal transferase dUTP nick-end labeling.

**Figure 4:**
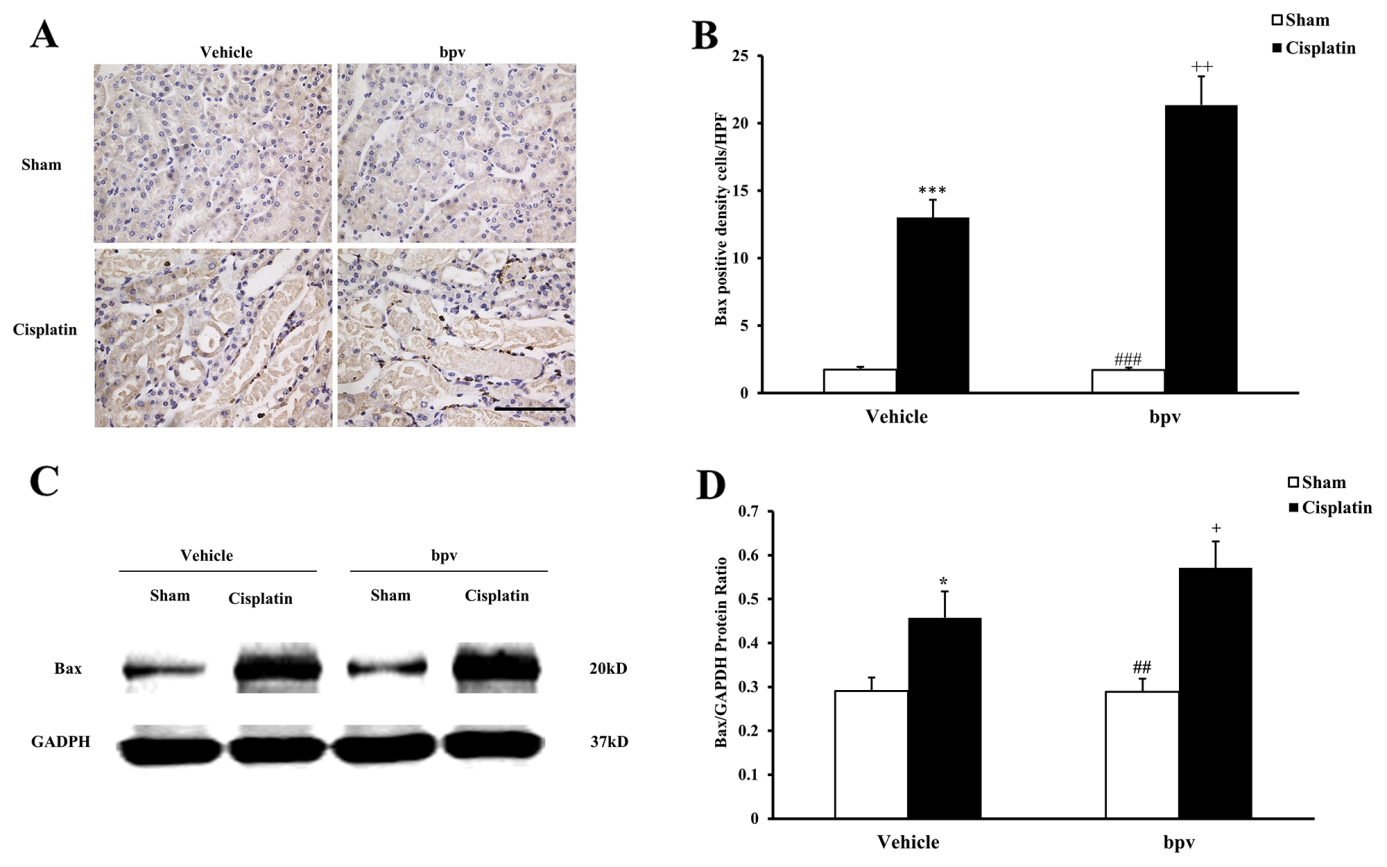
Inhibition of PTEN activity increased Bax protein expression in tubular epithelial cells from apoptosis in cisplatin-induced AKI **(A)** Representative photomicrographs of renal sections stained for Bax (brown) and counterstained with hematoxylin (blue) in vehicle and bpV(pic) administrated mice at 72h after sham or cisplatin treatment. (Original magnification: ×400). **(B)** Quantitative analysis of Bax expression in the kidneys of vehicle and bpV(pic) administrated mice after sham or cisplatin treatment. ***P < 0.001 vs. Vehicle Sham; ^+^P < 0.05 vs. Vehicle Cisplatin, ^###^P < 0.001 vs. bpV(pic) Cisplatin. n=6 each. **(C)** Representative western blots show Bax protein expression in the renal tissues from vehicle and bpV(pic) administrated mice at 72h after sham or cisplatin treatment. **(D)** Quantitative analysis of Bax protein expression in kidneys of vehicle and bpV(pic) administrated mice after sham or cisplatin treatment. ***P < 0.001 vs. Vehicle Sham; ^+^P < 0.05 vs. Vehicle Cisplatin, ^###^P < 0.001 vs. bpV(pic) Cisplatin. n=6 each.

**Figure 5:**
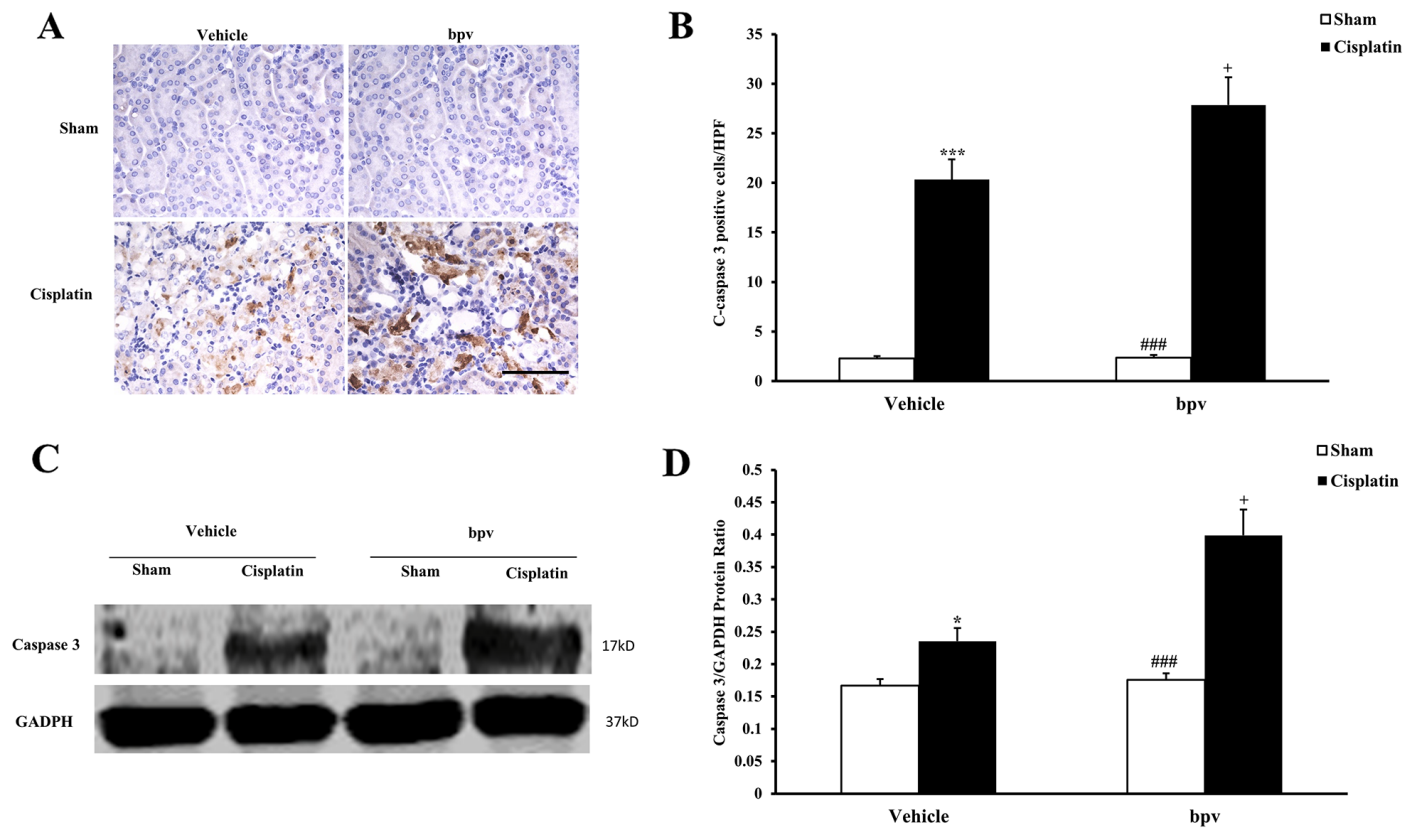
Inhibition of PTEN activity increased Cleaved Caspase 3 protein expression in tubular epithelial cells from apoptosis in cisplatin-induced AKI **(A)** Representative photomicrographs of renal sections stained for Cleaved Caspase 3 (brown) and counterstained with hematoxylin (blue) in vehicle and bpV(pic) administrated mice at 72h after sham or cisplatin treatment. (Original magnification: ×400). **(B)** Quantitative analysis of Cleaved Caspase 3 expression in the kidneys of vehicle and bpV(pic) administrated mice after sham or cisplatin treatment. ***P < 0.001 vs. Vehicle Sham; ^+^P < 0.05 vs. Vehicle Cisplatin, ^###^P < 0.001 vs. bpV(pic) Cisplatin. n=6 each. **(C)** Representative western blots show Cleaved Caspase 3 protein expression in the renal tissues from vehicle and bpV(pic) administrated mice at 72h after sham or cisplatin treatment. **(D)** Quantitative analysis of Cleaved Caspase 3 protein expression in kidneys of vehicle and bpV(pic) administrated mice after sham or cisplatin treatment. ***P < 0.001 vs. Vehicle Sham; ^+^P < 0.05 vs. Vehicle Cisplatin, ^###^P < 0.001 vs. bpV(pic) Cisplatin. n=6 each.

A growing body of evidence indicates that tubular cell apoptosis contributes to the pathogenesis of cisplatin-induced AKI [15, 16, 17]. Therefore, we examined the extent of cisplatin induced tubular epithelial cell apoptosis in both vehicle and bpV(pic) treated mice. Using a terminal transferase dUTP nick end labeling assay, we observed that the number of tubular apoptotic cells was significantly increased in the kidneys of vehicle mice receiving cisplatin treatment and markedly increased in the kidneys of bpV(pic) mice treated i.p. with cisplatin (Figure 3A and 3B). Bax is the pro-apoptotic member of the Bcl-2 family and determines the cytochrome c release and subsequent caspase activation, which induces a cell to apoptosis [18, 19]. Therefore, we evaluated the apoptosis of kidney tubule cells by Bax protein expression. Immunohistochemical staining (Figure 4A and 4B) and the western blotting analysis (Figure 4C and 4D) results showed that Bax expression was significantly higher in kidney tubular epithelial cells of vehicle mice after cisplatin treatment compared with sham controls, whereas Bax protein expression was markedly increased in the kidneys of bpV(pic)-treated mice compared with vehicle mice following cisplatin treatment. Caspases are crucial mediators of programmed cell death (apoptosis). Among them, caspase 3 is a frequently activated death protease and is essential for certain processes associated with the formation of apoptotic bodies [20, 21, 22]. Therefore, we next investigated the effect that PTEN activity has on caspase 3 protein expression in the pathogenesis of cisplatin-induced AKI with immunohistochemical staining (Figure 5A and 5B) and western blotting analysis (Figure 5C and 5D). The results revealed that caspase 3 was activated in kidney tubular epithelial cells of vehicle mice treated with cisplatin but that the levels of cleaved caspase 3 increased significantly in bpV(pic)-treated mice compared with vehicle mice following cisplatin treatment.

### Inhibition of PTEN activity increases inflammatory cell infiltration

**Figure 6:**
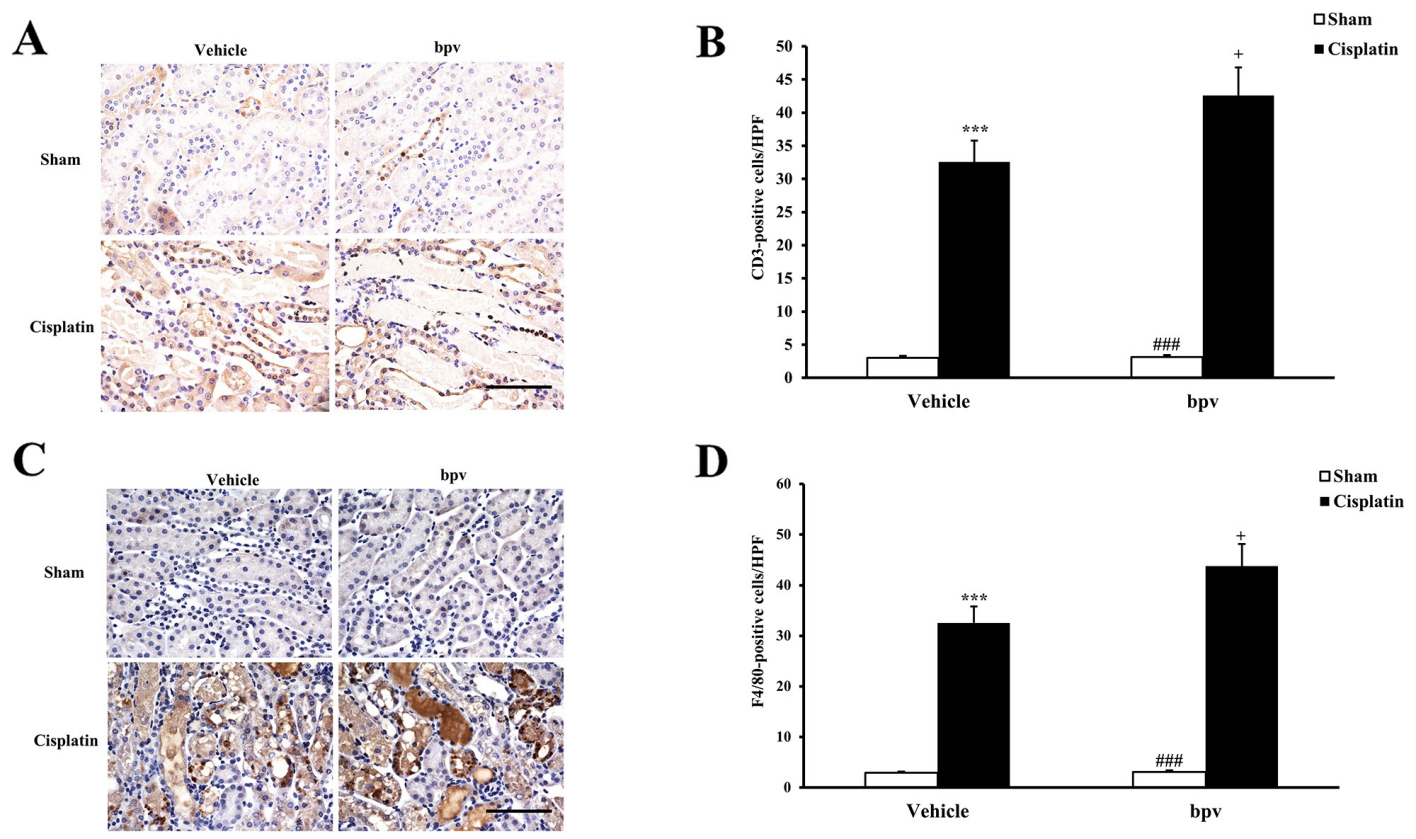
Inhibition of PTEN activity increased infiltration of T cells and phagocytes in the kidneys after cisplatin treatment **(A)** Representative photomicrographs of renal sections stained for CD3 (a T lymphocyte marker) (brown) and counterstained with hematoxylin (blue) in vehicle and bpV(pic) administrated mice at 72h after sham or cisplatin treatment. (Original magnification: ×400). **(B)** Quantitative analysis of CD3^+^ T cells in the kidneys from vehicle and bpV(pic) administrated mice at 72h after sham or cisplatin treatment. ***P<0.001 vs. Vehicle Sham; ^+^P<0.05 vs. Vehicle Cisplatin; ^###^P<0.001 vs. bpV(pic) Cisplatin. n=6 each. **(C)** Representative photomicrographs of renal sections stained for F4/80 (a phagocyte marker) (brown) and counterstained with hematoxylin (blue) in vehicle and bpV(pic) administrated mice at 72h after sham or cisplatin treatment. (Original magnification: ×400). **(D)** Quantitative analysis of F4/80^+^ phagocytes in the kidneys from vehicle and bpV(pic) administrated mice at 72h after sham or cisplatin treatment. ***P<0.001 vs. Vehicle Sham; ^+^P<0.05 vs. Vehicle Cisplatin; ^###^P<0.001 vs. bpV(pic) Cisplatin. n=6 each.

Inflammatory cells have an important role in the pathogenesis of cisplatin-induced AKI [23]. To examine whether the inhibition of PTEN activity affects the regulation of inflammatory cell infiltration into the kidney, kidney sections were stained for a lymphocyte marker (CD3) (Figure 6A and 6B) and a macrophage marker (F4/80) (Figure 6C and 6D). A significant infiltration of macrophages and T cells was observed in the kidneys of cisplatin-treated vehicle mice compared with vehicle sham controls. Inhibiting PTEN activity significantly increased the infiltration of macrophages and T cells into the kidneys after cisplatin treatment.

### Inhibition of PTEN activity increases inflammatory cytokine expression

**Figure 7:**
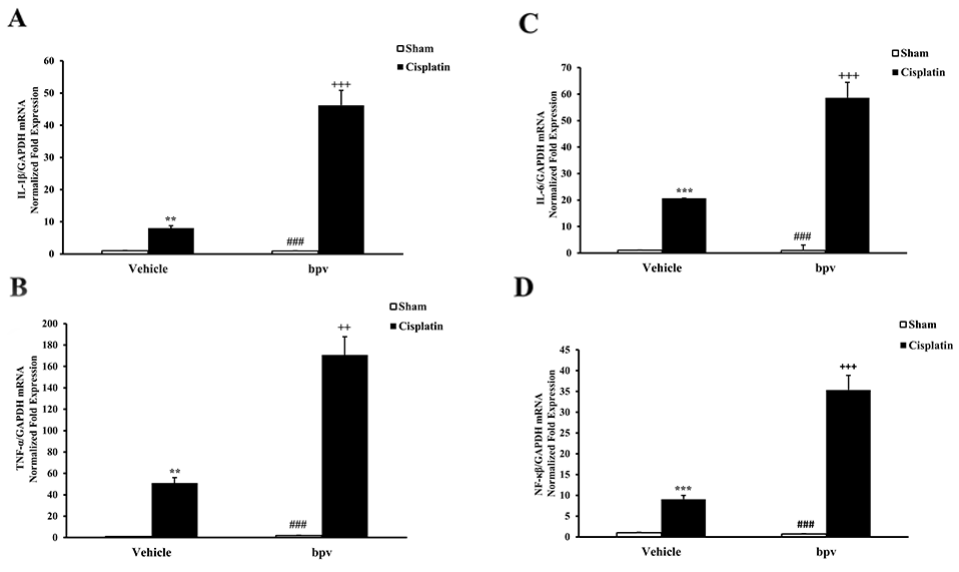
Inhibition of PTEN activity increased gene expression of proinflammatory molecules in the kidneys after cisplatin treatment **(A)** Quantitative analysis of IL-1β mRNA expression in the kidneys from vehicle and bpV(pic) administrated mice at 72h after sham or cisplatin treatment. ***P<0.001 vs. Vehicle Sham; ^+++^P<0.001 vs. Vehicle Cisplatin; ^###^P<0.001 vs. bpV(pic) Cisplatin. n=6 each. **(B)** Quantitative analysis of IL-6 mRNA expression in the kidneys from vehicle and bpV(pic) administrated mice at 72h after sham or cisplatin treatment. ***P<0.001 vs. Vehicle Sham; ^+++^P<0.001 vs. Vehicle Cisplatin; ^###^P<0.001 vs. bpV(pic) Cisplatin. n=6 each. **(C)** Quantitative analysis of TNF-α mRNA expression in the kidneys from vehicle and bpV(pic) administrated mice at 72h after sham or cisplatin treatment. ***P<0.001 vs. Vehicle Sham; ^+++^P<0.001 vs. Vehicle Cisplatin; ^###^P<0.001 vs. bpV(pic) Cisplatin. n=6 each. **(D)** Quantitative analysis of NF-κB mRNA expression in the kidneys from vehicle and bpV(pic) administrated mice at 72h after sham or cisplatin treatment. ***P<0.001 vs. Vehicle Sham; ^+++^P<0.001 vs. Vehicle Cisplatin; ^###^P<0.001 vs. bpV(pic) Cisplatin. n=6 each. GAPDH, glyceraldehyde-3-phosphate dehydrogenase.

To determine the role of PTEN activity on inflammatory cytokine expression in the pathogenesis of cisplatin-induced AKI, we examined the effect of bpV(pic) on the expression of the inflammatory cytokines involved in the pathogenesis of AKI by using real-time PCR. The results showed that the mRNA levels of IL-1β, IL-6, TNF-α and NP-κB in the kidney were significantly increased in the kidneys of vehicle mice after cisplatin treatment compared with sham controls and that the up-regulation of IL-1β, IL-6, TNF-α, and NP-κB was greatly increased in the kidneys of bpV(pic)-treated mice following cisplatin treatment (Figure 7).

### Inhibition of PTEN activity suppresses p53 phosphorylation in cisplatin-induced AKI

**Figure 8:**
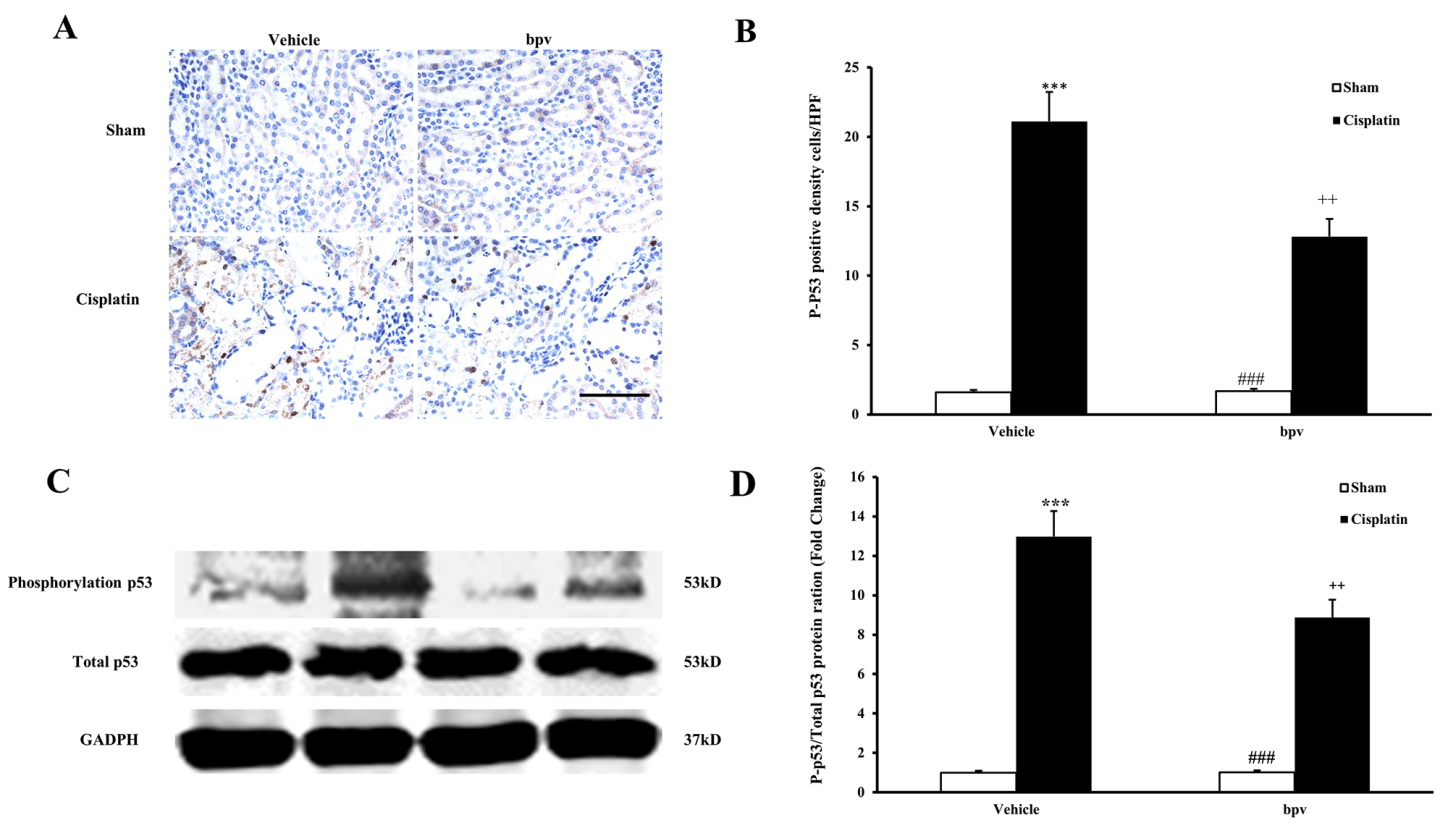
Inhibition of PTEN activity suppressed p53 phosphorylation in cisplatin-induced AKI **(A)** Representative photomicrographs of renal sections stained for phosphorylated p53 (brown) and counterstained with hematoxylin (blue) in vehicle and bpV(pic) administrated mice at 72h after sham or cisplatin treatment. (Original magnification: ×400). **(B)** Quantitative analysis of phosphorylation level of phosphorylated p53 in the kidneys from vehicle and bpV(pic) administrated mice at 72h after sham or cisplatin treatment. ***P<0.001 vs. Vehicle Sham; ^++^P<0.01 vs. Vehicle Cisplatin; ^###^P<0.001 vs. bpV(pic) Cisplatin. n=6 each. **(C)** Representative western blots showing phosphorylation level of p53 in the kidneys from vehicle and bpV(pic) administrated mice after sham or cisplatin treatment. **(D)** Quantitative analysis of phosphorylation level of p53 in the kidneys from vehicle and bpV(pic) administrated mice after sham or cisplatin treatment. ***P<0.001 vs. Vehicle Sham; ^++^P<0.01 vs. Vehicle Cisplatin; ^###^P<0.001 vs. bpV(pic) Cisplatin. n=6 each.

p53 is a transcription factor that has been demonstrated to be closely related to apoptosis [24]. Recent studies have suggested that the p53 pathway may also play a critical protective role in the pathogenesis of cisplatin-induced AKI [25, 26]. To explore the mechanisms of cell apoptosis that are associated with the inhibition of PTEN activity during the pathogenesis of cisplatin-induced AKI, we examined whether bpV(pic) affected the phosphorylation of p53 in cisplatin-induced AKI. Our results showed that cisplatin-induced AKI increased the number of cells that were positive for phosphorylated p53, as detected by immunohistochemical staining, in the kidneys of vehicle mice. The number of positive cells with phosphorylated p53 was significantly decreased in the kidneys of bpV(pic)-treated mice after cisplatin treatment compared with vehicle bpV(pic)-treated mice (Figure 8A and 8B). The western blotting results were consistent with these immunohistochemical staining findings (Figure 8C and 8D).

### Overexpression of PTEN alleviate apoptosis of renal tubular epithelial cell treated with cisplatin

**Figure 9:**
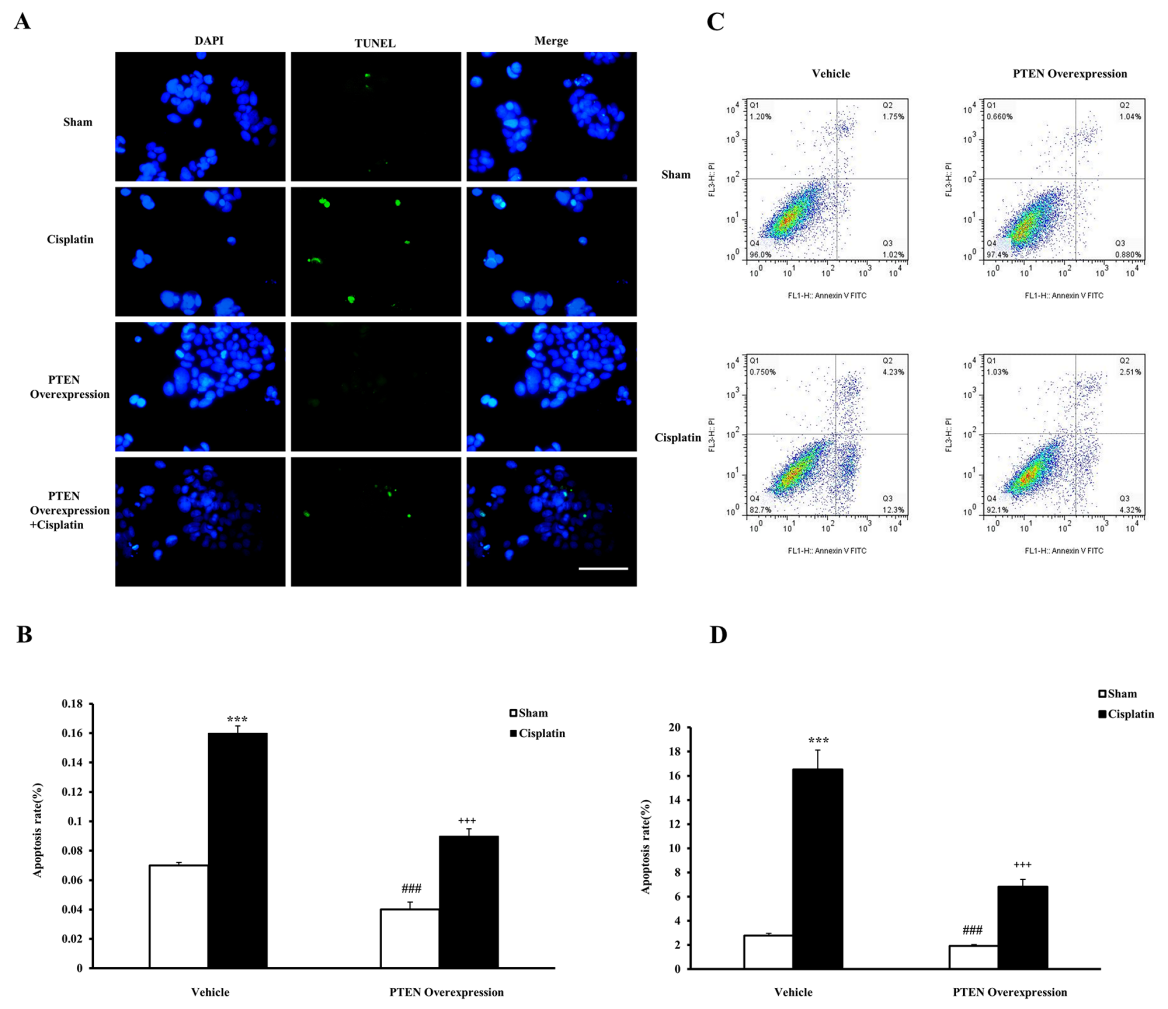
Effects of PTEN overexpression on cell apoptosis in mouse renal tubular epithelial cells **(A)** TUNEL assay of apoptosis with mouse renal tubular epithelial cells. **(B)** Quantitative analysis of apoptosis ratio from vehicle and PTEN overexpression administrated mice with sham or cisplatin treatment 6 hours. ***P<0.001 vs. Sham; ^++^P<0.001 vs. Cisplatin; ^###^P<0.001 vs. PTEN Overexpression Cisplatin. n=4 each. **(C)** Flow cytometry of apoptosis rate with mouse renal tubular epithelial cells. **(D)** The percentage of apoptotic cells from vehicle and PTEN overexpression administrated mice with sham or cisplatin treatment 6 hours. ***P<0.001 vs. Sham; ^+++^P<0.001 vs. Cisplatin; ^###^P<0.001 vs. PTEN Overexpression Cisplatin. n=4 each.

To strengthen our conclusion that the inhibition of PTEN activity aggravates cisplatin-induced AKI, we examined the effects that treating renal tubular epithelial cells with cisplatin *in vitro* and PTEN overexpression have on apoptosis. After renal tubular epithelial cells were transfected with the recombinant plasmids of PTEN, apoptosis of renal tubular epithelial cells was observed with cisplatin treatment using terminal transferase dUTP nick end labeling assay. The apoptosis rate of renal tubular epithelial cells was significantly increased in the cisplatin group compared with the sham group, and that of renal tubular epithelial cells was reduced in the PTEN overexpression cisplatin group compared with the cisplatin group (Figure 9A and 9B). Moreover, we detected the apoptosis cell ratio by using a flow cytometry analysis (Figure 9C and 9D), and the results were consistent with those obtained by TUNEL detection. Our results indicate that PTEN overexpression could induce the apoptosis of renal tubular epithelial cells.

## DISCUSSION

Cisplatin and other platinum derivatives are the most widely used chemotherapeutic agents to treat solid tumors such as head, ovarian, neck and lung cancers [27]. A common complication of cisplatin administration is AKI [28]. PTEN acts as a tumor suppressor by negatively regulating the PI3K signaling pathway, which plays an important role in AKI [29, 30, 31], and studies have indicated that PTEN is involved in the mechanism of AKI [8, 32]. However, the role of PTEN in cisplatin-induced AKI is unknown. In the present study, we have demonstrated the following: (1) The specific inhibition of PTEN activity exacerbated cisplatin-induced AKI and tubular cell apoptosis; (2) the specific inhibition of PTEN activity further activated caspase 3 and up-regulated the expression of Bax in cisplatin-induced AKI; (3) the specific inhibition of PTEN activity increased inflammatory cell infiltration and proinflammatory molecule production in the kidney following cisplatin treatment; and (4) the specific inhibition of PTEN activity suppressed the p53 signaling pathway, which is involved in cisplatin-induced AKI. These results indicated that PTEN, via the p53 signaling pathway, regulated inflammation and apoptosis and played a critical role in the pathogenesis of cisplatin-induced AKI.

A loss of function of the PTEN tumor suppressor, resulting in dis-regulated activation of PI3K signaling network, is recognized as one of the most common driving events in prostate cancer development [33, 34, 35]. PTEN has been increasingly investigated for its potential role in the pathophysiological process of acute or chronic injury of multiple organs [36, 37, 38]. Recent studies have emphasized that PTEN is a central regulator of various causes of AKI. A study by Potočnjak I suggested that the PTEN signaling pathway was involved in the pathogenesis of sepsis-induced AKI [8]. Sanghavi M proved that NFATc4 mediated by PTEN could prevent high-fructose-diet-induced renal injury [39]. It was reported that PTEN had important effects on ischemia reperfusion induced AKI [40]. Although many studies have investigated the role of PTEN in various types of AKI in recent years, little is known about the role of PTEN in cisplatin-induced AKI. In our study, we investigated the effect that PTEN has on the pathogenesis of cisplatin-induced AKI by using bpV(pic) (a PTEN-specific inhibitor). Our study shows that the inhibition of PTEN activity aggravates renal function after cisplatin-induced AKI.

It has often been reported that cisplatin induces both necrosis and apoptosis in renal tubular cells, depending on the dosage [41, 42]. Caspase 3 is formed from a 32 kDa zymogen that is cleaved into active 17 kDa subunits by death ligand and mitochondrial pathways. The activation of caspase 3 has been considered a key mechanism underlying the pathogenesis of cisplatin-induced apoptotic cell death in tubular epithelial cells [43]. As the first identified member of the Bcl-2 family, Bax is the key regulator of apoptosis at the molecular level [26]. Apoptosis is evaluated by terminal deoxynucleotidyl transferase-mediated digoxigenin-deoxyuridine nick-end labeling (TUNEL) assay of DNA damage, cleaved caspase 3 and Bax activation in present study. Our data showed that the number of TUNEL positive cells increased significantly in the kidneys of mice after cisplatin treatment and TUNEL positive cell increased markedly in bpV(pic)-treated mice in response to cisplatin treatment. Bax and caspase 3 are significantly activated in the kidneys of mice after cisplatin treatment, and Bax and caspase 3 activation are markedly up-regulated in kidneys of mice with PTEN activity suppression in response to cisplatin treatment. These data indicated that the inhibition of PTEN activity increased Bax and caspase 3 activation and apoptosis.

Inflammation plays an important role in cisplatin-induced AKI [28]. Inflammatory cells such as neutrophils and macrophages are known to infiltrate the kidney tissue and induce the release of proinflammatory cytokines [15]. Therefore, in the present study, we investigated the effect that PTEN activity has on cells staining positive for CD3 and F4/80 in the kidney after cisplatin treatment. Moreover, recent studies have shown that inflammatory cytokines contributed to the development of AKI [44, 45, 46]. Hence, we also examined the mRNA expression of IL-1β, IL-6, TNF-α and NF-κB in the kidney following cisplatin administration. Our results indicated that the inhibition of PTEN activity aggravated cisplatin-induced AKI by recruiting inflammatory cells into the kidney and increasing the expression of inflammatory cytokines.

It has been reported that p53 acts as a renoprotective agent after ischemic kidney injury by reducing inflammation [47]. It has also been reported that one of the molecular mechanisms of cisplatin-induced nephrotoxicity is involved in regulating the p53-dependent apoptosis pathway [48]. In our present study, we proved that the inhibition of PTEN activity increased cell apoptosis and activated inflammation reactions. Therefore, we speculated that the mechanism of PTEN activity in cisplatin-induced AKI was mediated by the p53 signaling pathway. In our study, we found that p53 phosphorylation was markedly increased in mice after cisplatin treatment compared with sham treatment, whereas p53 phosphorylation was markedly decreased in mice treated with bpV(pic) in response to cisplatin treatment. These results showed that PTEN was activated by cisplatin-induced AKI and played a protective role via the p53signaling pathway.

It is noteworthy that more specific studies focused on PTEN phosphorylation, especially micro RNA. Many studies have shown that reductive PTEN mediated by microRNAs such as miR-21, miR-106b, and miR-93 promoted cell progression via the PI3K/Akt pathway in various types of cancer [49, 50]. Recent evidence indicates that multiple microRNAs PTEN are involved in kidney development and the pathogenesis of kidney disease by targeting PTEN protein. For example, McClelland AD showed that miR-21 promotes renal fibrosis in diabetic nephropathy by targeting PTEN [51]. Another study reported that the blockade of miR-687 preserved PTEN expression and attenuated cell cycle activation and renal apoptosis, resulting in protection against kidney injury in mice [40]. Qingjuan L demonstrated that miR-148a-3p overexpression contributes to glomerular cell proliferation by downregulating PTEN in lupus nephritis [52]. However, although it is an important target spot for microRNA regulation, the functional role of PTEN in cisplatin-induced AKI is unknown. The results of the present study indicate that PTEN inhibition promotes kidney dysfunction in response to cisplatin, which lays the foundation for the further studies regarding the mechanism of micro RNA-mediated PTEN signaling pathway in cisplatin-induced AKI.

In summary, our study identified a fundamental effect of PTEN activity on cisplatin-induced AKI. In response to injury, the inhibition of PTEN activity suppressed the activation of the p53 signaling pathway, resulting in inflammatory cell infiltration and proinflammatory molecule production and cell apoptosis. These data indicated that regulating PTEN activity may constitute a novel therapeutic strategy for cisplatin-induced AKI.

## MATERIALS AND METHODS

### Animals

The animal experiments were conducted according to the guidelines of laboratory animal care and were approved by the Institutional Animal Care and Use Committee of the First People hospital of Foshan. BALB/c mice with bpV(pic) (Abcam, Cambridge, UK) treatment and vehicle controls received bpV(pic) (200 μg/kg) or saline i.p. every 4 hours, respectively. Implementation of the cisplatin-induced AKI model is described as follows: cisplatin was dissolved directly in 1 mg/ml of 0.9% saline. Male BALB/c mice, 8 to 12 weeks of age and weighing approximately 20 to 30 grams, were administered cisplatin (20 mg/kg) or saline by i.p, injection. Animals were sacrificed at 72 h after cisplatin injection.

### Measurement of renal function

Serum creatinine was measured using a creatinine assay kit (BioAssay Systems, Hayward, CA, USA) according to the manufacturer’s instruction. Blood urea nitrogen was determined fluorometrically as described [53].

### PTEN activity

The PTEN Activity ELISA is designed to quantify the phosphatase activity of PTEN by detecting the product, PIP2, in a competitive ELISA format, thus eliminating the need for radioactivity, organic solvents, and thin layer chromatography. PTEN activity was detected using the experimental procedure according to the assay kit’s instruction (K-4700-1kit, Echelon, USA).

### Renal morphology

Kidney tissue was fixed in 10% buffered formalin, embedded in paraffin, and cut to 4 μm thickness. After deparaffinization and rehydration, sections were stained with hematoxylin and eosin. Tissue damage was examined in a blinded manner and scored according to the percentage of damaged tubules: 0, no damage; 1, less than 25% damage; 2, 25%–50% damage; 3, 50%–75% damage; and 4, more than 75% damage as reported [54].

### Immunohistochemistry

Immunohistochemical staining was performed on paraffin sections. Antigen retrieval was performed with antigen unmasking solution (Vector Laboratories) or proteinase K. Endogenous peroxidase activity was quenched with 3% H_2_O_2_ for 10 min. After blocking with 5% normal serum, slides were incubated with primary antibodies in a humidified chamber overnight. After washing, slides were sequentially incubated with appropriate secondary antibodies and ABC solution according to the ABC kit (Vector Laboratories). Slides were then visualized by incubation in diaminobenzidine solution for an appropriate time duration. Nuclear staining was performed with hematoxylin. The slides were dehydrated, cleared, and mounted. The images from these slides were obtained and analyzed using the NIS Element software (Nikon Instruments) with a Nikon microscope image system (Nikon Instruments).

### Detection of apoptotic cells in renal sections

Apoptotic cell death was determined by using terminal deoxynucleotidyl transferase-mediated dUTP nick-end labeling (TUNEL) staining with the DeadEnd Colorimetric Apoptosis Detection System (Millipore, Billerica, MA, USA) according to the manufacturer’s instruction. The number of TUNEL-positive cells per high-power field was counted and analyzed in a blinded fashion.

### Quantitative real-time RT-PCR

Total RNA was extracted from kidney tissues with TRIzol reagent (Invitrogen). Aliquots (1 μg) of total RNA were reverse transcribed using SuperScript II reverse transcriptase. Real-time PCR was performed using IQ SYBR green supermix reagent (Bio-Rad, Herculus, CA) with a Bio-Rad real-time PCR machine according to the manufacturer’s instructions. The comparative Ct method (ΔΔCt) was used to quantify gene expression, and the relative quantification was calculated as 2^−ΔΔCt^. The expression levels of targeted genes were normalized to GAPDH level in each sample. The primer sequences were as follows:

IL-1β-forward, 5’ CTCGGCCAAGACAGGTCGCTC 3’
-reverse, 5’ CCCCCACACGTTGACAGCTAGG 3’
IL-6-forward, 5’ AGGATACCACTCCCAACAGACCTG-3’,
-reverse, 5’ CTGCAAGTGCATCATCGTTGTTCA-3’;
TNF-α-forward, 5’ CATGAGCACAGAAAGCATGATCCG-3’,
-reverse, 5’ AAGCAGGAATGAGAAGAGGCTGAG-3’;
NF-κB-forward, 5’ GAGACATCCTTCCGCAAACT 3’
-reverse, 5’ TCCTTCCTGCCCATAATCA 3’
GAPDH-forward, 5’ CCAATGTGTCCGTCGCGTGGATCT-3’,
-reverse, 5’ GTTGAAGTCGCAGGAGACAACC-3’.

### Western blot analysis

Protein was extracted using RIPA buffer containing cocktail proteinase inhibitors and quantified with a Bio-Rad protein assay. An equal amount of protein was separated on SDS-polycrylamide gels in Tris/SDS buffer system, and then transferred onto nitrocellulose membranes. Blotting was performed according to standard procedures with primary antibodies of Bax, cleaved caspase 3, and p53 overnight, followed by incubation with appropriate fluorescence-conjugated secondary antibodies. The proteins of interest were analyzed using an Odyssey IR scanner (LI-COR Biosciences), and the signal intensities were quantified using the NIH Image/J software (National Institutes of Health).

### Cell culture and experimental grouping

Mouse Renal Tubular Epithelial Cells were thawed in a 38°C water bath and then centrifuged at 1000 rpm for 5 min. The supernatant was discarded, and the cells were cultured in DMEM medium in an incubator containing 5% CO2 at 37°C. The medium was replaced when cells adhered to the bottle wall. The cells were subcultured until the cells covered 80% of the bottle bottom. The cells were divided into four groups: sham group, cisplatin group, PTEN overexpression group and PTEN overexpression cisplatin group. Cells were treated with cisplatin 10 μg/ml or sham control 6 h in each group.

### Plasmid construction and cell transfection

The overexpression plasmid of the PTEN gene (pcDNA3.1+PTEN) was manufactured by Mingshanshang Medical Biotechnology Co., Ltd. (Guangzhou City, P.R. China). It was verified by PCR, restriction enzyme digestion, and DNA sequencing. Mouse renal tubular epithelial cells were seeded into 96-well plates (100 μL/well). Then, the cell lines were transfected with recombinant plasmids (pcDNA3.1+PTEN) using Lipofectamine 2000 and incubated in an incubator containing 5% CO2 at 37°C for 6 hours. Next, cells were cultured with fresh medium containing 10% fetal bovine serum (FBS) for 48 h.

### Statistical analysis

Data were presented as the means of ± SEM. Multiple groups of comparison were performed by ANOVA followed by the Bonferroni procedure. A P value < 0.05 was considered statistically significant.
